# Significant Differences in Host-Pathogen Interactions Between Murine and Human Whole Blood

**DOI:** 10.3389/fimmu.2020.565869

**Published:** 2021-01-15

**Authors:** Silke Machata, Sravya Sreekantapuram, Kerstin Hünniger, Oliver Kurzai, Christine Dunker, Katja Schubert, Wibke Krüger, Bianca Schulze-Richter, Cornelia Speth, Günter Rambach, Ilse D. Jacobsen

**Affiliations:** ^1^ Research Group Microbial Immunology, Leibniz Institute for Natural Product Research and Infection Biology, Hans Knoell Institute, Jena, Germany; ^2^ Research Group Fungal Septomics, Leibniz Institute for Natural Product Research and Infection Biology, Hans Knoell Institute, Jena, Germany; ^3^ Institute for Hygiene and Microbiology, University of Würzburg, Würzburg, Germany; ^4^ Institute of Hygiene and Medical Microbiology, Medical University of Innsbruck, Innsbruck, Austria; ^5^ Institute of Microbiology, Friedrich Schiller University Jena, Jena, Germany

**Keywords:** whole blood ex vivo model, host-pathogen interaction, *Candida albicans*, neutrophils, mice

## Abstract

Murine infection models are widely used to study systemic candidiasis caused by *C. albicans*. Whole-blood models can help to elucidate host-pathogens interactions and have been used for several *Candida* species in human blood. We adapted the human whole-blood model to murine blood. Unlike human blood, murine blood was unable to reduce fungal burden and more substantial filamentation of *C. albicans* was observed. This coincided with less fungal association with leukocytes, especially neutrophils. The lower neutrophil number in murine blood only partially explains insufficient infection and filamentation control, as spiking with murine neutrophils had only limited effects on fungal killing. Furthermore, increased fungal survival is not mediated by enhanced filamentation, as a filament-deficient mutant was likewise not eliminated. We also observed host-dependent differences for interaction of platelets with *C. albicans*, showing enhanced platelet aggregation, adhesion and activation in murine blood. For human blood, opsonization was shown to decrease platelet interaction suggesting that complement factors interfere with fungus-to-platelet binding. Our results reveal substantial differences between murine and human whole-blood models infected with *C. albicans* and thereby demonstrate limitations in the translatability of this *ex vivo* model between hosts.

## Introduction

Dissemination of pathogens from a primary site of colonization or infection can occur *via* different routes, including lymphatic vessels and the blood stream (1). The hematogenous is by far the most frequent route for systemic infections of various bacterial and fungal pathogens, in the most severe cases leading to blood stream infections (2). Survival in blood can thus be considered a major virulence trait in the development of systemic infections. However, our understanding of how pathogens interact with cellular and humoral host factors in blood is limited, mainly due to technical issues: While it is relatively easy to study the interaction of pathogens with isolated blood cells, or their survival in serum or plasma, such approaches lack the complexity of interactions between different types of immune cells and additional factors, e.g., complement, present in blood. Assessing host-pathogen interactions *in vivo* in patients is challenging due to ethical and logistic limitations. In mice, as the most commonly used laboratory animal for *in vivo* experiments, the blood volume that can be withdrawn repeatedly is very limited and thereby hampers in depth analysis of interactions within blood. Furthermore, if bacteremia or fungemia occurs transiently or intermittingly, pathogens might not be detectable in every blood sample during hematogenous dissemination (3). Therefore, we previously established an *ex vivo* human whole-blood infection model that allowed us to define which immune cells interact with the human fungal pathogen *Candida albicans* (4), to identify cross-talk between different components of the host response (5), and to detect substantial differences between related fungal pathogens (6).

*Candida* infections (candidiasis) caused by *C. albicans* commonly arise from endogenous strains that colonize mucosal surfaces as a commensal in healthy individuals. Risk factors for candidiasis are microbiota imbalance, impaired mucosal barrier function, and immunosuppression (7, 8). In the majority of cases the fungus causes relatively benign mucosal infections such as oral and vaginal candidiasis (9). Life-threatening infections arise from dissemination *via* the blood stream resulting in deep-seated or systemic candidiasis (8). Disseminated candidiasis is associated with high mortality rates that can exceed 50% despite antifungal therapy (10, 11). Dissemination from the gut into internal organs can be triggered in mice by a combination of intestinal barrier disruption and immunosuppression (12), but the most commonly used model to study systemic candidiasis is intravenous infection of mice (13). This model is considered a gold standard tool for detailed investigations of fungal virulence and of host immune responses, and largely resembles catheter-associated disseminated candidiasis in men (13, 14). However, the initial interaction of *C. albicans* with murine blood and its impact on the development of systemic candidiasis is not well understood. Following intravenous infection, *C. albicans* rapidly disappears from circulating peripheral blood (15, 16), yet it is unknown whether this is due to killing of circulating fungal cells, adhesion to endothelium, rapid invasion of internal organs, or a combination of these factors.

In the human whole-blood model, neutrophils predominantly associate with and phagocytose *C. albicans* (4). Fungal killing in the human whole-blood model is to 98% accountable to neutrophils (4) and mediated by phagocytosis, degranulation and formation of extracellular traps. However, murine blood differs significantly from human blood in the abundance of neutrophils: These cells are the most abundant leukocytes in human blood (55–70%) while murine blood is dominated by lymphocytes (70–80%) with neutrophils accounting only for 8% to 24% of all leukocytes (17). To elucidate whether these differences affect interaction of *C. albicans* with whole blood, we adapted our protocol established for human blood for application to mice. Our results show significant differences between humans and mice in the *ex vivo* model, including lower association of the fungus with leukocytes, higher association with thrombocytes, and substantially less killing of *C. albicans* in murine blood.

## Materials and Methods

### Ethics Statement

Human peripheral blood was collected from healthy volunteers with written informed consent. This study was conducted in accordance with the Declaration of Helsinki and all protocols were approved by the Ethics Committee of the University Hospital Jena (permit number: 273-12/09). All animals used for the project were held in accordance with the European Convention for the Protection of Vertebrate Animals Used for Experimental and Other Scientific Purposes and the experiments performed in accordance with European and German regulations. The sacrifice of mice for blood withdrawal was performed under §4 “removal of organs” of the German Animal Welfare Act and was approved by the local animal welfare officer (no specific permit number issued). The systemic candidiasis model was approved by the Thuringian authority and ethics committee (Thüringer Landesamt für Verbraucherschutz, permit number 03-007/13 and HKI-19-003).

### Strains and Culture Conditions

*C. albicans* GFP-expressing strains M137 (18) (pACT1-GFP) and *C. albicans* Δ*efg1*Δ*cph1* + pADH1-GFP were used. To construct *C. albicans* Δ*efg1*Δ*cph1* + pADH1-GFP a CaGFP-caSAT1 construct with homology regions for the integration into the *C. albicans ADH1* locus was excised with *Asc*I/*Sac*I from the plasmid pSK-ADH1prom-CaGFP-SAT1 (4) and then transformed into the *EFG1*/*CPH1* double mutant HLC52 (19). Transformation was performed with the established lithium acetate protocol (20). Transformants were grown for two days on YPD with 200 mg/ml nourseothricine and verified by PCR and microscopy. For experiments *C. albicans* over-night cultures grown in YPD medium (2% peptone, 1% yeast extract, 2% glucose) at 30°C and 180 rpm were diluted 1:50 in YPD medium and sub-cultured for another 3 to 4 h into the mid-log phase under the same conditions. Yeast cells were washed three times with PBS, counted and diluted in PBS to 5 × 10^7^/ml or 5 × 10^6^/ml, as indicated.

GFP-expressing *S. aureus* [6850/pALC1743 (21, 22)] and *Escherichia coli* ATCC 25922 (23) were cultivated overnight at 37°C, 180 rpm in LB medium. The overnight culture was inoculated 1:100 into fresh LB medium and incubated at 37°C, 180 rpm until O.D_600_ 0.6 to 0.7 was reached. The cultures were then washed three times with PBS and bacterial cell numbers were calculated based on OD_600_–CFU correlations. Cultures were diluted to desired concentrations with PBS before inoculation of whole blood.

### Whole-Blood Model

Human peripheral blood from healthy donors was collected in hirudin-monovettes (Sarstedt, Germany, 2.7 ml volume). Hirudin was used as an anti-coagulant as it was previously shown to have no effect on complement activation (24). Due to their size, direct use of the hirudin-monovettes was not feasible for the collection of murine blood. Therefore, three hirudin-monovettes were rinsed with total volume of 500 µl sterile saline (0.9% NaCl) to solubilize the hirudin. Needles and syringes rinsed with hirudin-NaCl were then used to collected blood from female BALB/c mice (8–12 weeks old, Charles River, Germany) or from C57BL/6J mice (8–12 weeks old, Service Unit Experimental Biomedicine, Friedrich-Schiller-University Jena, Germany) by heart puncture immediately after sacrifice by intraperitoneal application of an overdose of ketamine (500 mg/kg) and xylazin (25 mg/kg). The blood was then immediately transferred to a falcon tube containing 500 µl hirudin-NaCl, in which blood from ten animals was pooled for each experiment. Pilot experiments analyzing fungal CFU in whole blood of female and male C57BL/6J mice revealed no sex-specific differences; thus, both male and female mice were used for the experiments.

The human whole-blood infection assay was performed as described previously (4), and murine blood was infected in the same way. Briefly, 1/50 volume fungal or bacterial suspensions prepared as described above were added to murine or human blood, resulting in an infectious dose of 1 × 10^6^/ml for most experiments or 1 × 10^5^/ml for lower infection dose experiments. The incubation of the whole blood was carried out at 37°C under slow constant motion on a rolling device (Phoenix instrument RS_TR05) for time points from 10 min to 480 min. Survival of the pathogens was determined by plating on agar plates (YPD for *C. albicans*, LB for *E. coli* and *S. aureus*) using serial dilutions in PBS and counting colony forming units. To determine the effect of ketamine/xylazin on phagocytosis and association of *C. albicans* with immune cells, 40 µg/ml ketamine and 2 µg/ml xylazin were added to human blood directly before the experiment. 40 µg/ml ketamine is equivalent to fivefold the maximal serum concentration in mice after intraperitoneal application of 100 mg/kg ketamine (25).

### Blood Analysis and Flow Cytometry

Generally hematology analysis of murine samples was performed using a BC-5300Vet (Mindray) configured for murine blood. For murine samples, blood smears were prepared for each time point and stained by May-Gruenwald-Giemsa staining (Roth). Filamentation and interaction with platelets were observed by light microscopy. Further analyses were performed by differential staining and subsequent measurement with a flow cytometer. For murine blood, differential staining was performed with CD45-PerCP (Cat. no. 557235, clone 30-F11, 12 µg/ml, BD Biosciences), CD19-APC-Cy7 (Cat. no. 115530, clone 1D3; 12 µg/ml, Biolegend) for B-cells, CD3e-V500 (Cat. no. 560771, clone 500A2, 12 µg/ml, BD Biosciences) for T-cells, Ly6G-PE (Cat. no. 127608, clone 1A8, 12 µg/ml final conc; Biolegend) for neutrophils, NK1.1-BV421 (for C57BL/6J mice: Cat. no. 108731, clone PK136, 12 µg/ml, Biolegend) or CD335-BV421 (for BALB/C mice: Cat. no. 562850, Clone 29A1.4, 12 µg/ml, BD Biosciences) for NK cells, CD11b-APC (Cat. no. 130-091-241, clone M1/70.15.11.5, 30 µg/ml, Miltenyi) for monocytes and neutrophils, CD41 (Cat. no. 133906, clone MWReg30, 12 µg/ml, Biolegend) for platelets, and CD69-PE vio770 (Cat. no. 130-103-944, clone H1.2F3, 30 µg/ml, Miltenyi) and CD62-PE-Vio779 (Cat. no. 130-105-537, clone REA 344, 30 µg/ml, Miltenyi) as platelet activation markers. In parallel, staining with the appropriate isotype controls (APC Rat IgG2b, κ Isotype Control, clone RTK5430, Cat. no. 400611, Biolegend; V500: Syrian hamster IgG2 κ Isotype Control, clone B81-3, Cat. no. 560785, BD Bioscience; PE: rat IgG2a κ Isotype Control, clone RTK2758, Cat. no. 400507, Biolegend; PerCP rat IgG2b κ Isotype Control, clone A95-1, Cat. no. 552991, BD Bioscience; APC-Cy7 rat IgG2a κ Isotype Control, clone RTK2758, Cat. no. 400524, Biolegend; V450 rat IgG2a κ Isotype Control, clone R35-95, Cat. no. 560377, BD Bioscience; V450 mouse IgG2a κ Isotype Control, clone G155-178, Cat. no. 560550, BD Bioscience; PE Vio770 hamster IgG1 Isotype Control, clone G235-2356, Cat. no. 553956, BD Bioscience; PE rat IgG1 κ Isotype Control, clone RTK2071, Cat. no. 400407, Biolegend; APC human IgG1 Isotype Control, clone QA16A12, Cat. no. 403505, Biolegend) was performed as a binding specificity control. After staining the erythrocytes were removed using the BD FACS Lysing solution and fixed samples were analyzed on a FACSVerse (BD Biosciences) flow cytometer after two washing steps in PBS supplemented with 3% FCS.

Human whole-blood was analyzed with a BD FACSCanto II flow cytometer. Platelets were specifically identified by CD42b^+^ staining (mouse anti-human CD42b-APC antibody, clone HIP-1, Cat. no. 303912, BioLegend). Co-staining with mouse anti-human CD66b-V450 antibody (clone G10F5, Cat. no. 561649, BD Bioscience) identified platelets associated with neutrophils. Activation of human platelets was investigated by changes in surface CD62P expression (mouse anti-human CD62P-PE antibody, clone AK-4, Cat. no. 555524, BD Bioscience). In parallel, staining with the appropriate isotype controls (APC mouse IgG1, κ Isotype Ctrl, clone MOPC-21, Cat. no. 400122, BioLegend; V450 mouse IgM, κ Isotype Control, clone G155-228, Cat. no. 560861, BD Bioscience and PE Mouse IgG1, κ Isotype Control, clone MOPC-21, Cat. no. 555749, BD Bioscience) was performed as a binding specificity control. Stained blood samples were treated with BD FACS Lysing solution followed by washing and harvesting cells in BD CellWASH solution. FlowJo10 was used for analysis of all samples.

### Neutrophil Isolation From Murine Bone Marrow

Neutrophils were isolated from bone marrow of femur and tibia of three to four female 8- to 12-week-old BALB/c mice as previously described (26). Briefly, bone marrow was flushed with RPMI 1640 supplemented with Penicillin/Streptomycin (Sigma) and homogenized using a 40 µm cell strainer. Lysis of erythrocytes was carried out for 1 min on ice using a lysis buffer containing 8 mg/ml NH_4_Cl, 1 mg/ml K_2_CO_3_ and 0.01% EDTA. Neutrophils were purified using a Percoll gradient of 52%, 69%, and 78% in PBS and cells were collected from the 69% to 78% interface. After washing with HBSS neutrophils were resuspended in HBSS, analyzed on a BC-5300Vet (Mindray) and stored on ice until usage for a maximum of 1 h. The purity of isolated neutrophils was roughly 70%.

### Analysis of Platelets From Mice With Systemic Candidiasis

Six- to eight-week-old female speciﬁc-pathogen-free BALB/c mice (16 to 18 g) purchased from Charles River (Germany) were housed in groups of five in individually ventilated cages with free access to food and water. Mice were infected with 2.5 × 10^4^ *C. albicans* CFU/g body weight in 100 µl DPBS *via* the lateral tail vein at day 0. Groups of mice were sacrificed at the indicated time points post infection by intraperitoneal application of an overdose of ketamin (500 mg/kg) and xylazin (25 mg/kg). 100 µl of blood were collected under terminal anesthesia by retro-orbital bleeding, immediately transferred into a tube containing 10 µl EDTA solution (1.6 mg/ml), and gently mixed. 20 µl of the sample were analyzed on a BC-5300Vet (Mindray) to determine platelet counts and mean platelet volume. Platelet-rich plasma (RPP) was prepared from 40 µl of whole-blood by centrifugation at 135 g for 15 min at room temperature. To detect platelet activation, platelets were stained for 30 min with fluorescence-labeled antibodies (BioLegend) directed against CD41 (clone HIP8, Cat. no. 303710, 0.1 µg/ml) as platelet marker and CD63 (clone H5C6, Cat. no. 353006, 8 µg/ml) as activation marker, followed by fixation with 1% formaldehyde. Surface expression of CD62P, fibrinogen binding and C3c binding were determined as described for CD63 using fluorescence-labeled antibodies (CD62P: BioLegend, clone AK4, Cat. no. 304906, 1.5 µg/ml; fibrinogen: BioRad, polyclonal, Cat. no. 4440-8004F, 100 µg/ml; C3c: Dako, polyclonal, Cat. no. F0201, 400 µg/ml). Non-activated unstained cells and non-activated and activated stained cells were used to calibrate the flow cytometer using single stains and combined stains. Plasma was prepared by centrifugation of 40 µl whole-blood at 1500 g for 15 min at room temperature. Plasma concentrations of soluble CD62P were determined using the Quantikine^®^ ELISA Mouse sP-Selectin/CD62P kit (R&D Systems, USA) performed according to manufacturer instructions.

### Isolation of Platelets and Confrontation Assay

Venous blood of healthy human volunteers was collected in sodium citrate monovettes (Sarstedt) and directly centrifuged at 80 × g for 20 min (without break). Supernatants were collected and treated with 0.25 mg/ml acetylsalicylic acid (Sigma Aldrich) for 30 min followed by addition of 1 mM Prostaglandin E1 (Sigma Aldrich), both to prevent pre-activation of the containing platelets. Centrifugation at 400 × g for 8 min pelleted platelets that were afterwards suspended in HEPES-Tyrodes buffer (10 mM HEPES, 137 mM NaCl, 2.8 mM KCl, 1 mM MgCl_2_6H_2_O, 12 mM NaHCO_3_, 0.4 mM Na_2_HPO_4_2H_2_O, 5.5 mM Glucose, 0.35% BSA, without Ca^2+^). Purified platelets were treated again with 1 mM Prostaglandin E1, centrifuged at 400 × g for 10 min and suspended either in RPMI 1640 medium containing 20% autologous active or heat-inactivated plasma. Autologous human plasma was collected after centrifugation of Hirudin-anticoagulated blood from the same donor at 16,000 × g for 10 min. To inactivate complement proteins, autologous human plasma was incubated for 1 h at 56°C. Murine platelets were prepared from freshly collected blood of healthy mice as concentrates by thrombocytapheresis with Amicus cell separator (Baxter, Vienna, Austria) by the Department of Immunology and Blood Transfusion (Innsbruck Medical University, Innsbruck, Austria). The platelet concentration was determined by a hemocytometer and adjusted to a concentration of 1.2 to 1.4 × 10^9^/ml. Murine serum was collected by centrifugation of blood samples from (i) SPF mice without any treatment, (ii) mice systemically infected with a sublethal dose of 1 × 10^4^ *C. albicans* CFU/g body weight on days 8 to 21 after infection (see 2.6), (iii) C3-deficient mice (B6.129S4-C3^tm1Crr^/J). Pooled serum of 5 to 12 mice per group was used for experiments with isolated platelets.

Confrontation of isolated platelets with *C. albicans* was performed at a ratio of platelets to fungal cells of 10:1 for the indicated time points (37°C, constant rolling at 5 rpm). Mock-infected platelets with the two different media served as controls.

### Quantification of Cytokines

Plasma samples were generated from whole-blood aliquots that were incubated on ice for 45 min to 1 h and subsequently centrifuged at 4°C for 10 min at 10,000 × g. Supernatants were stored at −80°C until further use. Concentration of IFN-γ, IL-1β, TNF-α, IL-6, and KC (CXCL1) in murine samples was determined by ELISA (Invitrogen, Thermo Fisher Scientific) performed according to manufacturer’s instructions.

## Results

### *C. albicans* Survives in Murine Whole Blood

**Figure 1:**
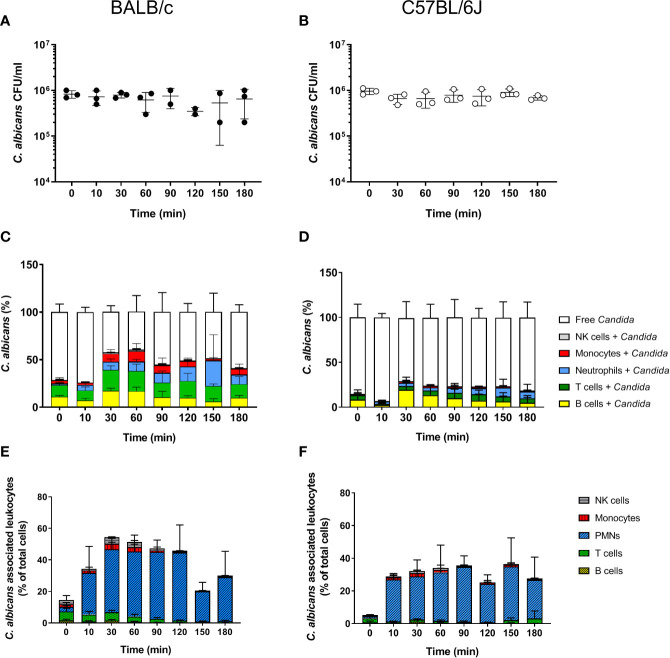
Survival and association of *C. albicans* with leukocytes in murine whole blood. Colony forming units (CFU) were determined from infected blood of BALB/c **(A)** and C57BL/6J **(B)** mice. Each dot represents the data from an independent experiment; lines represent mean ± standard deviation (n = 3, except BALB/c 90 min and 120 min: n = 2). **(C, D)** The percentage of GFP-expressing *Candida* binding to immune cells of blood was calculated relative to total *C. albicans* cells in infected blood (set to 100%) during a time course of 3 h. All values correspond to the means of three independent experiments with pooled whole blood from 10 mice. Left: BALB/c; right: C57BL/6. **(E, F)** The percentage of various immune cells associated with *Candida* was determined relative to their overall cell population in the blood. All values correspond to the means of three independent experiments with pooled whole blood from 10 mice. Left: BALB/c; right: C57BL/6.

The fate of *C. albicans* upon exposure to murine blood was analyzed in whole blood collected from two of the most commonly used mouse strains for infection experiments, BALB/c and C57BL/6J. As described by Hünniger et al. for the human whole-blood model (4), whole blood was inoculated with 1 × 10^6^/ml of yeast-grown *C. albicans* and fungal survival was determined by counting colony forming units (CFU) at various time points over a time course of 3 h. In contrast to human blood in which a 50% decrease of the initial CFUs within the first hour of contact was observed (4), the fungal burden did not decrease during incubation in neither BALB/c nor C57BL/6J mouse blood but remained stable throughout the experiment (**Figures 1A, B**). Of note, ketamine and xylazin were used to euthanize mice prior to blood collection. As ketamine has been shown to affect antimicrobial functions of macrophages and neutrophils in a dose-dependent manner (27–30), we added ketamine and xylazin to human blood and quantified fungal killing. At the used dose (40 µg/ml ketamine and 2 µg/ml xylazin) ketamine/xylazin treatment did not affect fungal killing (**Figure S1**).

### Less Immune Cells Interact With *C. albicans* in Murine Compared to Human Blood

In order to determine whether the higher fungal survival in murine compared to human blood was associated with differences in the interaction with immune cells, we analyzed the number of immune cells and association of *C. albicans* with leukocytes. Quantification of total leukocyte numbers using a hematology analyzer showed a moderate, non-significant decline over time in both mouse strains (**Figure S2A**). While the numbers of CD45^+^ leukocytes in relation to all blood cells showed no obvious changes throughout the infection (**Figure S2B**), the numbers of neutrophils decreased by almost 50% within the first 10 min after addition of *C. albicans* (**Figure S2C**). This was not the case for mock-infected control samples (**Figure S2D**). In contrast, the number of other types of leukocytes remained stable over time (**Figure S3**). Note that the relative abundance of neutrophils was significantly lower in the blood of B57BL/6J (4.56 ± 1.3%) than of BALB/c mice (8.63 ± 1.58%; *p* = 0.02).

Using a strain constitutively expressing green fluorescent protein the association of *C. albicans* with leukocytes was determined by flow cytometry. Within 30 min, more than 50% of *C. albicans* cells were associated with immune cells in BALB/c whole blood (**Figure 1C**). The proportion of fungal cells associated with immune cells remained relatively stable until the end of the experiments (**Figure 1C**). A similar trend was observed in blood of C57BL/6J mice; however, the rate of association was lower and did not exceed 40%. Surprisingly, a large proportion of *C. albicans* cells were found to be associated with B- and T-cells in both mouse lines (**Figures 1C, D**). This contrasts results from the human whole-blood model in which monocytes and neutrophils were the dominant types of immune cells physically interacting with *C. albicans* (4). Since the ratio of the different immune cell populations in blood differs significantly between humans and mice, we speculated that the association to lymphocytes could be the consequence of the higher abundance of these types of immune cells in murine blood rather than the result of specific interactions. We thus calculated the percentage of host cells interacting with *C. albicans* within the different immune cell populations (**Figures 1E, F**). Although only a small fraction of *C. albicans* cells were associated with neutrophils, up to 50% and 40% of all neutrophils associated to fungal cells in blood from BALB/c and C57BL/6J mice, respectively. In contrast, only a minor fraction of the B- and T-cell populations interacted with the fungus, suggesting that the observed association is indeed a stochastic physical event rather than the result of specific interactions.

### The Reduced Fungal Killing in Murine Blood Is Only Partially Due to Lower Neutrophil Numbers

**Figure 2:**
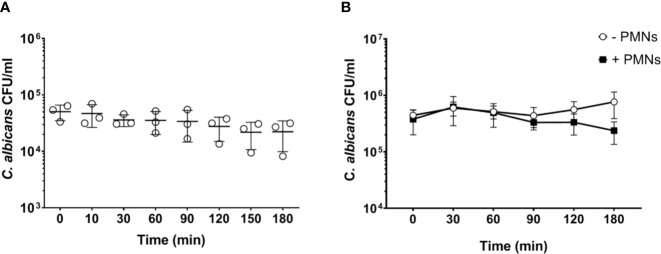
Increased PMN - to - *Candida* ratio leads to a non-significant decrease of the fungal burden after 180 min. **(A)** Colony forming units were determined on YPD plates from infected blood samples at various time points for 3 h. All values correspond to the means of three independent experiments with pooled whole blood from 10 BALB/c mice infected *ex vivo* with 1 × 10^5^/ml of *C. albicans::gfp*. Each dot represents the mean of an independent experiment ± standard deviation (n = 3). **(B)** Colony forming units were counted in murine whole blood as described for **(A)** with the following modifications: the infection dose was 1 × 10^6^/ml and murine whole blood was supplemented with 4.5 × 10^6^/ml neutrophils that were previously isolated from bone marrow of BALB/c mice. Although differences between control and neutrophil supplemented blood were observed at later time points, these were not statistically significant (p>0.05, Wilcoxon matched-pairs signed rank test).

Neutrophils are the most active immune cells interacting with *C. albicans* in both human and murine blood, and have been shown to be critical for killing of the fungus in the human whole-blood model (4). However, the absolute number of neutrophils is approximately 10-fold lower in murine (0.4 × 10^6^/ml) than human blood (4 × 10^6^/ml; according to reference values and determined by a hematology analyzer). Using the same infection dose (1 × 10^6/^ml) in both models thus resulted in a pathogen to neutrophil ratio of 2.5 in murine but 0.25 in human blood. We hypothesized that the higher microbe to neutrophil ratio in the murine whole-blood model overwhelmed the capacity of the neutrophils to interact with and control *C. albicans*. Therefore, we analyzed fungal survival in whole blood of BALB/c mice using a 10-fold lower infection dose (1 × 10^5/^ml), which is comparable to the ratio used previously in human whole blood (4). With the reduced infection dose there was a moderate reduction of the fungal burden by 30% within 3 h of infection (**Figure 2A**). A similar effect was observed when murine blood was supplemented with neutrophils isolated from bone marrow to reach cell numbers comparable to human blood (4.4 ± 0.51 × 10^9^/L of cells, determined by a hematology analyzer) (**Figure 2B**). Thus, both the addition of external neutrophils and the lower infection dose led to increased fungal killing, but survival of *C. albicans* after 180 min was still substantially higher (>40%) than in a human whole-blood model (10% (4);). Furthermore, fungal burden decreased only slowly in murine blood, whereas 50% of fungal cells were killed within the first 60 min in human blood (4). To exclude effects mediated by ketamine/xylazin, ketamine and xylazin were added to human blood; this did not affect association rates with neutrophils and monocytes (**Figure S4**).

### Filamentation of *C. albicans* in Whole Blood Is Not Effectively Inhibited by Murine Neutrophils but Fungal Survival Does Not Require Filamentation

**Figure 3:**
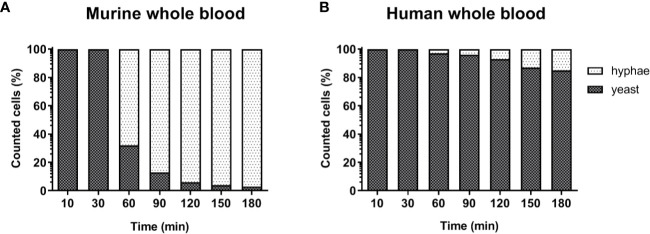
Morphology of *C. albicans* in murine and human blood during the course of infection. Blood smears were prepared at the given time points and the state of fungal filamentation was determined by counting yeast or hyphae form of *Candida* (n = 100 per time point) on an Axioplan 2 microscope (Zeiss) after staining of cells with Mai-Gruenwald- Giemsa.

The lower number of neutrophils in murine compared to human blood did not fully explain the lower killing of *C. albicans* in murine blood. However, functional differences between human and murine neutrophils have been described: A previous study from Ermert et al. (31) described a reduced efficiency of murine neutrophils to kill *Candida* species due to the fact that mice lack β-defensins and produce lower amounts of myeloperoxidase. They also showed that internalized *C. albicans* can escape from murine but not from human neutrophils by outgrowing neutrophils through filamentation and subsequent rupture of the neutrophil membrane (31). We therefore analyzed *C. albicans* morphology in whole blood by microscopy. Following infection with yeast cells, after 60 min the majority of *C. albicans* (~70%) in murine blood were hyphae and after 180 min nearly all (>90%) fungal cells grew as filaments (**Figure 3A**). In contrast, hyphal morphology was observed only for 10% and 20% of all *C. albicans* cells in human blood after 60 and 180 min, respectively (**Figure 3B**).

**Figure 4:**
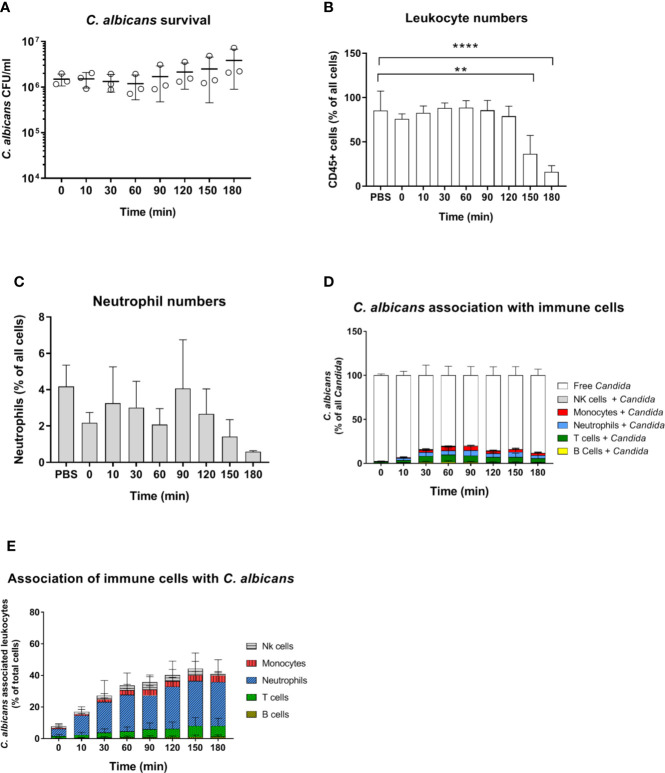
Survival and immune cell association of the yeast-locked *C. albicans* strain Δ*efg1*Δ*cph1* + pADH1-GFP in murine whole-blood of BALB/c mice. **(A)** Whole blood was pooled from 10 mice and infected *ex vivo* with 1 × 10^6^/ml of the GFP-expressing *C. albicans* mutant. CFU were determined from infected blood at indicated time points. Each dot represents the mean of an independent experiment ± standard deviation (n = 3). **(B**, **C)** Immune cell stability during the course of infection, **(B)** relative number CD45^+^ cells and **(C)** neutrophils as % of all cells. Asterisks indicate significant differences (**p<0.005, ****p<0.0005; 1-Way ANOVA and Holm-Sidak’s multiple comparison test to compare each infected time point to 0 min). **(D)** The percentage of GFP-expressing *Candida* binding to immune cells was calculated relative to total *C. albicans* cells in infected blood (set to 100%) during a time course of 3 h. All values correspond to the means of three independent experiments with pooled whole blood from 10 mice. **(E)** The percentage of various immune cells associated with *Candida* was determined relative to their overall cell population in the blood. All values correspond to the means of three independent experiments with pooled whole blood from 10 mice.

*C. albicans* morphology has been shown to affect recognition by neutrophils (5, 32) and monocytes (33). Furthermore, while human neutrophils prevent *C. albicans* filamentation and escape following phagocytosis (31), human monocytes and macrophages do not, and intracellular filamentation leads to immune cell lysis and fungal escape (34). Given the higher rate of *C. albicans* filamentation observed in murine blood, and the role of morphology for immune escape, we used a yeast-locked *C. albicans* Δ*efg1*/Δ*cph1* mutant expressing GFP (19) in the murine whole-blood infection model to determine if the higher filamentation rate could explain higher survival of *C. albicans* in murine blood. Surprisingly, no decrease in CFU counts was observed with this strain and fungal numbers even increased 2.5-fold from 60 to 180 min (**Figure 4A**). The increasing fungal burden correlated with a decrease in CD45^+^ cells (**Figure 4B**), and especially neutrophils (**Figure 4C**), at later time points (150 min and 180 min). The percentage of *C. albicans* Δ*efg1*/Δ*cph1* cells not associated with any of type of immune cell (~80%, **Figure 4D**) was significantly higher compared to the wild type strain (~50%, **Figure S2A**). This is in contrast to experiments with human blood, in which similar association of wild type and mutant with immune cells was observed (4, 5). However, more than 25% of all neutrophils were associated with this mutant after 2 h (**Figure 4E**), and the release of cytokines upon infection with the mutant was comparable to or higher than *C. albicans* wild type infection (**Figure S4**). Thus, the large number of free Δ*efg1*/Δ*cph1* cells cannot be explained by an inability of immune cells to recognize mutant yeast cells.

### Rapid Platelet Aggregation and Activation in Murine Blood Upon Exposure to *C. albicans*

**Figure 5:**
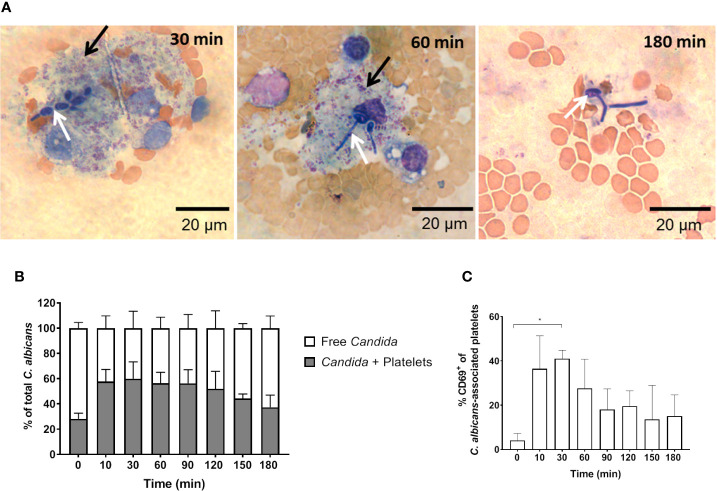
*C*. *albicans*-induced platelet activation and aggregation **(A)** Fungal interactions with platelets were visualized in blood smears of *ex vivo* infected murine whole blood after staining with Mai-Gruenwald- Giemsa. Aggregates of thrombocytes (black arrow) surround *Candida* (white arrow) mostly during the first hour of infection. Flow cytometry analysis shows **(B)** strong binding of *C. albicans* to platelets almost immediately after infection and **(C)** induced expression of CD69 on the surface of platelets. Asterisks indicate significant differences (*p*<0.05; 1-Way ANOVA and Holm-Sidak’s multiple comparison test to compare each infected time point to 0 min).

**Figure 6:**
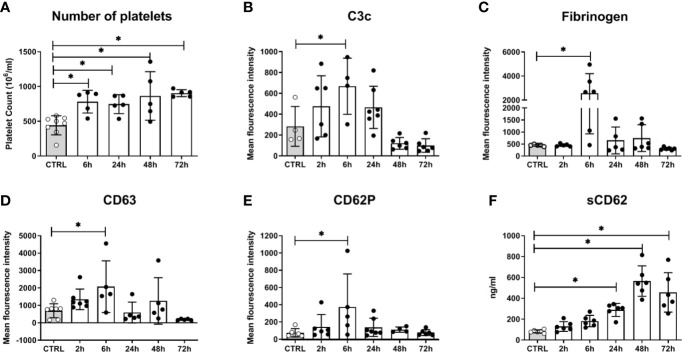
Platelet activation in mice systemically infected with *C. albicans*. **(A)** The number of circulating platelets in peripheral murine blood was determined with an automated hematology analyzer. Deposition of complement factor C3 **(B)**, fibrinogen **(C)**, and surface expression of CD63 **(D)** and CD62P **(E)** was quantified by flow cytometry. **(F)** Soluble CD62 was measured in plasma by ELISA. CTRL: Samples from non-infected control animals. Time on X axis: Time after infection. Four to seven samples were analyzed per time point, data is presented as individual data points with bars indicating the mean ± standard deviation. Asterisks indicate significant differences (*p*<0.05; 1-Way ANOVA and Holm-Sidak’s multiple comparison test to compare each infected group to the control).

In addition to fungal filamentation, microscopic analysis revealed aggregates of platelets around *C. albicans* cells (**Figure 5A**). This interaction was confirmed and quantified by flow cytometry (**Figure 5B**): Within 10 min almost 60% of *C. albicans* cells were associated with platelets. The association rate slowly decreased over time to 40% after 3 h. At early time points, over 35% of the fungal-associated platelets expressed the activation marker CD69 on their surface (**Figure 5C**). We also observed increased expression of CD62 on the surface of *C. albicans* bound platelets, however in only 20% of all platelets (**Supplementary Figure S6**). To exclude that platelet activation was an artefact of the *ex vivo* situation, blood samples of intravenously (i. v.) infected mice were analyzed. *In vivo*, platelet counts increased significantly over a course of three days post infection (**Figure 6A**). Transient platelet activation was observed at 6 h post infection characterized by a significant increase of complement C3c, indicating opsonization, the platelet aggregation factor fibrinogen, and the activation markers CD63 and CD62P on the surface of platelets (**Figures 6B–E**). While at later time points expression of these markers was comparable to control samples, increased concentrations of soluble CD62 were detectable in the plasma of infected mice from 1 to 3 days post infection by ELISA (**Figure 6F**). Taken together, these data demonstrate interaction and activation of platelets following infection with *C. albicans* in murine blood *ex vivo* and *in vivo*.

### Opsonization of *C. albicans* in Human Blood Leads to Low Platelet Association

**Figure 7:**
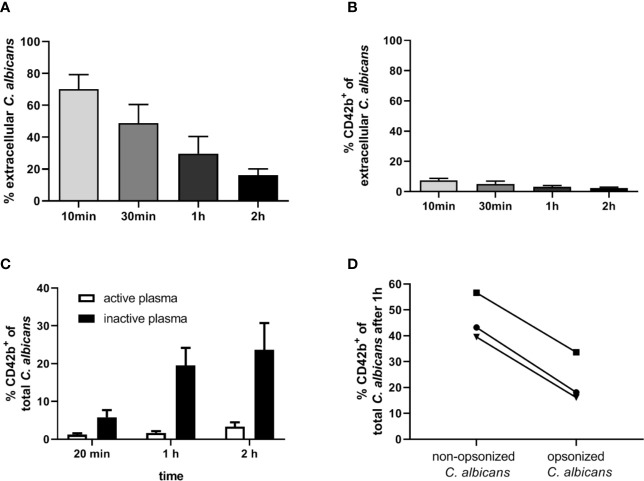
Interaction of human platelets with *C. albicans* during whole-blood infection is influenced by complement opsonization. **(A)** The percentage of extracellular *C. albicans* during human whole-blood infection was investigated *via* flow cytometry and calculated relative to total fungal cells in blood (set to 100%) of five independent experiments with blood from different donors. A steady reduction of extracellular fungi over time was observed (1-Way ANOVA and Test for linear trend, *p*<0.0001). **(B)** Less than 10% of extracellular *C. albicans* cells showed a CD42b^+^ staining at each time point, reflecting a low platelet binding to *C*. *albicans* in human whole-blood (n = 5). **(C)** Isolated human platelets were confronted with *C. albicans* either in the presence of non-treated (active plasma) or heat-inactivated autologous plasma (inactive plasma). Co-incubation in medium containing active plasma prevented the time-dependent increase in platelet-*C. albicans* interaction as observed for heat-inactivated plasma. Significantly less CD42b-positive fungal cells were observed with active compared to inactive plasma at each time point (unpaired, two-tailed t-test, p<0.0001, n = 7). **(D)** *C. albicans* cells were either pre-incubated in non-treated (C3b-opsonized *C. albicans*) or heat-inactivated autologous plasma (non-opsonized *C. albicans*, no surface C3b) before confrontation with isolated human platelets in medium with inactive plasma for 1 h. Opsonization of *C. albicans* resulted in a markedly lower association with platelets compared non-opsonized *C. albicans* (*p* = 0.035, unpaired, two-tailed t-test).

**Figure 8:**
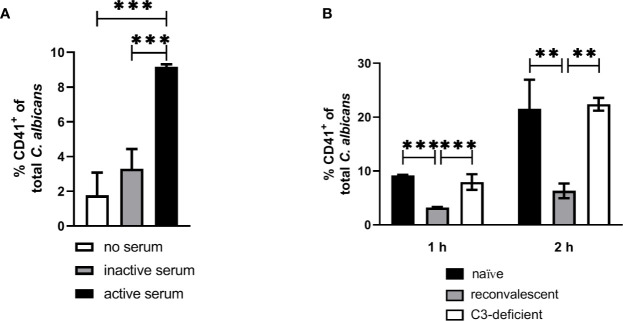
Interaction of isolated murine platelets with *C. albicans*. **(A)** Binding of isolated platelets to *C. albicans* cells in the presence of heat-inactivated serum (inactive serum) or non-treated serum (active serum). The percentage of CD41-positive C. albicans cells was determined after 1 h by flow cytometry. Active serum led to a significantly higher interaction with platelets compared inactive serum and a control without serum (asterisks indicate significant differences (*p*<0.05) determined by 1-Way ANOVA and Holm-Sidak’s multiple comparison test for pairwise comparisons of all groups). **(B)** Binding of isolated platelets to *C. albicans* cells in the presence of active serum from naïve mice, serum of mice collected after infection with *C. albicans* (reconvalescent) and serum from naïve C3-deficient mice analyzed after 1 h and 2 h. Reconvalescent serum led to significantly lower binding (significance determined by 1-Way ANOVA and Holm-Sidak’s multiple comparison test for pairwise comparisons of all groups within one time point and indicated by asterisks).

Our next aim was to determine if interaction with thrombocytes also occurs in human blood. Consistent with previous findings (4), we observed decreasing numbers of *C. albicans* cells not associated with host cells in human blood over time (**Figure 7A**). Only a small proportion (less than 10%) of fungal cells directly associated with platelets (**Figure 7B**). This was in sharp contrast to the results we obtained in murine blood. To investigate the potential influence of human blood components on fungal-platelet interaction, confrontation of isolated human platelets with *C. albicans* cells was performed in the presence of non-treated (active) or heat-inactivated autologous plasma and showed a time-dependent increase in platelet-*C. albicans* interaction when plasma proteins were inactivated (**Figure 7C**). In contrast, the presence of non-treated plasma containing active complement proteins resulted in a low platelet association. Consequently, we tested if opsonization of fungal cells interferes with platelet binding. Indeed, pre-opsonized *C. albicans* showed a significantly reduced interaction with isolated platelets compared to non-opsonized *C. albicans* (**Figure 7D**) indicating that opsonization of extracellular *C. albicans* during human whole-blood infection prevents the binding of platelets. In a comparable experiment using isolated murine thrombocytes and murine serum, active but not inactive serum significantly increased the binding of thrombocytes to *C. albicans* (**Figure 8A**).

The observation that inactivation of human plasma increases interaction of thrombocytes with *C. albicans* suggests that complement factors interfere with binding of human platelets to the fungus. While it remains unclear how complement becomes activated in active human plasma upon contact with *C. albicans*, the classical, antibody-mediated complement activation pathway might be involved. Specific-pathogen free mice, as used in this study, are usually not colonized by *C. albicans* (35) and show comparatively low colonization with fungi (36), resulting in very low detectable anti-*Candida* antibodies in SPF mice (37). Thus, due to the absence of antibodies, whole murine blood and serum might lack the level of opsonization mediated by normal human plasma and, in consequence, lower opsonization could result in higher interaction with platelets. To test this hypothesis, we compared the binding of isolated thrombocytes to *C albicans* in the presence of active serum from normal SPF mice (naïve mice) and mice that survived a sublethal systemic *C. albicans* infection (reconvalescent mice). The binding of thrombocytes to fungal cells was significantly higher with serum from naïve mice; furthermore, naïve serum and serum from C3-deficient mice induced similar binding of thrombocytes to *C. albicans* (**Figure 8B**), supporting the hypothesis that the lack of prior contact to the fungus in SPF mice contributes to the observed differences between human and murine whole blood regarding interaction with thrombocytes.

### Whole Murine Blood Is Not Able to Clear Bacterial Infections *Ex Vivo*

**Figure 9:**
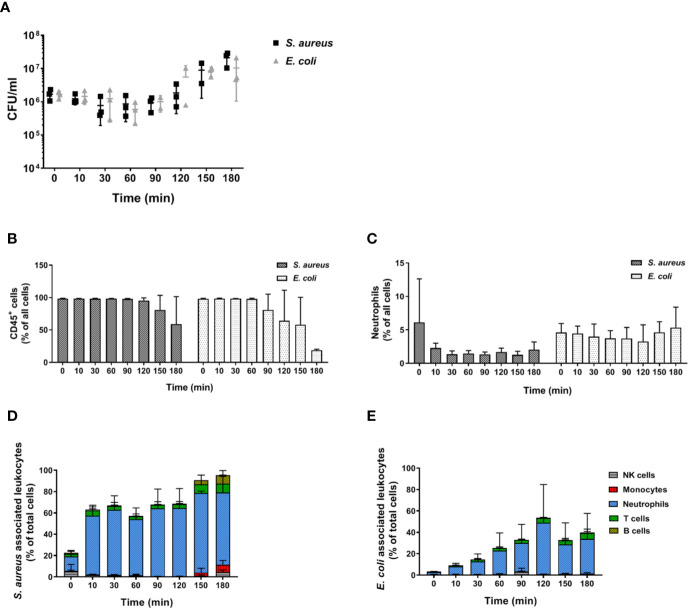
Bacterial survival and immune cell association in murine whole blood. **(A)** Colony forming units of *S. aureus* and *E. coli* were determined from infected murine blood. All values correspond to the means of three independent experiments with pooled whole-blood from 10 BALB/c mice infected *ex vivo* with 1 × 10^6^/ml of GFP-expressing bacteria. Each dot represents the mean of an independent experiment +/- standard deviation (n = 3). The relative numbers of CD45^+^ cells **(B)** and of **(C)** neutrophils (Ly6G^+^/CD11b^+^) during the course of infection were measured by flow cytometry. **(D)** The percentage of various immune cells associated with GFP-expressing *S. aureus* or **(E)** with GFP-expressing *E. coli* was determined relative to their overall cell population in the blood. All values correspond to the means of three independent experiments with pooled whole blood from 10 mice.

In order to determine whether the inability of murine blood to control infections *ex vivo* is restricted to *C. albicans*, we performed infections with *S. aureus* and *E. coli* as models for Gram-positive and Gram-negative bacteria, respectively. Similar to our observations with the filament-deficient *C. albicans* strain, CFU counts for both bacterial pathogens remained stable for the first 90 min followed by a steady increase (**Figure 9A**). This increase in pathogen load was even more pronounced than that observed for *C. albicans* Δ*efg1* Δ*cph1* (**Figure 4A**), and was also seen with a 10-fold lower infection dose, albeit at a lesser extent (**Figure S7**). Also similar to infection with the yeast-locked *C. albicans* mutant was the reduction of leukocyte numbers (**Figure 9B**), and for *S. aureus* especially neutrophil counts (**Figure 9C**), at late time points. The association of bacteria to immune cells varied quite drastically between both bacterial strains: While almost 60% of neutrophils were rapidly bound to *S. aureus* within 10 min and almost all neutrophils were attached to these Gram-positive bacteria 1.5 h post infection (**Figure 9D**), bacterial host cell association occurred much later for *E. coli* and a maximum of 50% of neutrophils were attached to bacteria at 2 h post infection (**Figure 9E**).

## Discussion

Intravenous infection of mice is the most commonly used model to study candidiasis (13). It is known that *C. albicans* disappears quickly from circulating blood within a few minutes to hours after infection (15, 16, 38), but the mechanisms underlying this phenomenon and the fungal interaction with blood components are not fully understood. We have previously employed a human whole-blood model to determine the interaction of *C. albicans* with leukocytes and the fate of the fungus. In this study, the model was adapted to mice to elucidate host-fungal interactions in whole blood over a period of 3 h.

One of the most striking differences observed between the murine and human whole-blood experiments was the lack of *C. albicans* killing in murine blood, both at the infectious dose used in our previous study on human blood (1 × 10^6^/ml) and at a 10-fold lower dose (1 x 10^5^/ml). A commonly used infectious dose in the intravenous mouse model is 2.5 × 10^4^ CFU/g body weight, equivalent to 5 × 10^5^ CFU for a 20 g mouse (13, 16). The approximate blood volume of a 20 g mouse is 1.6 ml (39), resulting in approximately 3 × 10^5^ CFU injected per ml of blood. Thus, the doses used in this study are relevant for the *in vivo* mouse model. Consequently, the inability of murine blood to effectively eliminate the fungus cannot be explained by an inadequately high infectious dose. Furthermore, as comparable results were obtained with blood from BALB/c and C57BL/6 mice, low fungal killing is unlikely to be a consequence of the specific genetic background.

Differences were also observed in the interaction with immune cells: A higher proportion of fungal cells was not associated to any leukocytes in murine (40–50%) compared to human blood (10–20% (4),). Especially the proportion of *C. albicans* cells associated with neutrophils was substantially lower in mouse blood (<30% compared to >60% (4),). Neutrophils are known to be the major effector cells mediating *C. albicans* killing in human whole-blood infection (4), and they are essential for host defense against disseminated candidiasis both in humans (40, 41) and mice (42, 43). It is unlikely that the low number of *C. albicans* interacting with neutrophils is due to insufficient recognition, as the high proportion of neutrophils associated with *C. albicans* indicated that these leukocytes responded to the fungus. The lower absolute numbers, however, lead to a pathogen-to-neutrophil ratio that might have been too high to allow efficient infection control. Contrary to this hypothesis, reducing the infectious dose did not substantially increase fungal killing. It is possible that the abundance of other immune cells blocked physical contact between neutrophils and *Candida*. Consistent with this, many fungal cells were associated with lymphocytes during high dose infection, even though the proportion of lymphocytes interacting with *Candida* was low, indicating limited directed activity. We therefore increased the absolute number of murine neutrophils to that in human blood, thereby also altering the neutrophil-lymphocyte ratio. As this had only a minor impact on fungal killing, functional differences between murine and human immune cells are likely to contribute to fungal survival in murine blood.

While human and murine neutrophils share many characteristics, they differ in some functional aspects. For example, murine neutrophils do not produce defensins (44), express less myeloperoxidase and lysozyme (45), and differ in the expression and production of pattern recognition receptors, cytokines, and chemokines [summarized in (46)]. Ermert et al. showed that this translates into functional differences: Isolated murine neutrophils were less efficient in killing *C. albicans in vitro* than their human counterparts (31). Furthermore, while neutrophils were shown to be responsible for preventing hyphae development in human blood (18), isolated murine neutrophils fail to inhibit filamentation of phagocytosed yeast, resulting in fungal escape (31). Consistent with this, we observed substantial filamentation of *C. albicans* in murine, but not human blood. Thus, the higher filamentation rate in murine blood might be the consequence of an inability of murine neutrophils to control filamentation not only *in vitro* but also in the more complex *ex vivo* model. Killing of neutrophils by *C. albicans* filamentation after phagocytosis would furthermore explain the decline of neutrophil numbers over time in infected murine blood. However, extracellular filamentation has been shown to also occur in human blood (4), and we can therefore not exclude that the increased filamentation observed in murine blood is due to the lower number of fungal cells interacting with immune cells.

Although long filaments are more difficult to phagocytose due to their size (47), it is unlikely that filamentation alone is responsible for the lack of fungal killing, as survival and even proliferation in murine whole blood was observed for a filament-deficient *C. albicans* mutant. This was surprising because mutants lacking either of the genes (*EFG1* and *CPH1*) deleted in the mutant used here were previously reported to display reduced survival in human blood (18), and the double mutant is avirulent in a systemic mouse infection model (48). The association rate of this mutant with leukocytes was however lower compared to wild type fungi (<25% compared to up to 50%). This is indicative of lower phagocytosis rates and likely leads to less phagocytosis-dependent fungal killing. The lower association rates could be due to the generally less efficient recognition of yeast *versus* hyphal cells by neutrophils (32, 49). The specific gene deletions furthermore affect the exposure of cell wall components serving as pathogen-associated molecular patterns (50–52). As the percentage of neutrophils interacting with the non-filamentous mutant was however only slightly less than for the wild type, it appears unlikely that altered recognition alone is responsible for proliferation of the filament-deficient mutant. This is supported by the cytokine measurements that demonstrate similar or higher responses of murine whole blood to the mutant compared to the wildtype, and is consistent with the higher cytokine release by monocytes and PBMCs in response to yeast cells observed by others (33, 53).

While our results overall suggest a very limited capacity of murine neutrophils to control *C. albicans* in murine blood, it should be noted that we observed a reduction of fungal burden at late time points in murine whole blood with added neutrophils. Although not statistically significant, this is in agreement with other studies demonstrating some efficacy of isolated murine neutrophils against this fungus (31, 54). In this context it should be noted that neutrophils are primed during recruitment to a site of inflammation, leading to increased expression of pattern recognition receptors and enhanced effector functions (55, 56). Thus, the limited impact of neutrophils in murine whole blood cannot be transferred to neutrophil efficacy in tissue *in vivo*. However, our results suggest that the rapid decline of *C. albicans* CFU numbers in the peripheral blood of intravenously infected mice is not due to fungal killing within circulating blood. Possibly, interaction between *C. albicans* and murine blood leukocytes *in vivo* occurs only after fungal attachment to endothelial cells (57, 58).

Host-dependent differences in the interaction of *C. albicans* with leukocytes were also observed for platelets: In the murine whole-blood model, aggregation of platelets around and adhesion to fungal cells was observed which was associated with platelet activation. Transient activation of platelets was also observed *in vivo* in mice, consistent with previous observations by others (38). In contrast, only few platelets associated with *C. albicans* in human blood, supporting recent findings by Eberl et al. (59). The absence of the formation of obvious platelet aggregates around fungal elements in human blood is consistent with other studies (60, 61), and might be mediated by fungal chitin (62). Importantly, others showed that only those human platelets that bound to *C. albicans* became activated (59). The low number of bound platelets likely results in a very small increase of activation in the overall platelet population. This could explain why several studies described that this fungus does not activate human platelets (60, 63, 64), whereas we detected a significant increase of released platelet effector molecules. Interestingly, we found that the physical interaction between *C. albicans* and human platelets was enhanced by plasma inactivation, suggesting that complement factors interfere with binding of human platelets to the fungus. In this context our observation that prior exposure of mice to *C. albicans* significantly affected the impact of their serum on the rate of interaction between the fungus and thrombocytes might be of relevance not only for this, but also for other studies and might have implications beyond *C. albicans* infection: SPF mice, which are commonly used to study infections, lack contact to common opportunistic pathogens and thus adaptive immune response to these microbes. As demonstrated here, this could explain some of the differences observed between mice and men, and might also contribute to the limited efficacy of murine whole blood to eliminate *E. coli* and *S. aureus* shown in this study. Even though *S. aureus* has been isolated from laboratory SPF mice colonies, over 70% of tested animals were found to be free of this opportunistic pathogen (65). It can also be assumed that SPF mouse colonies are free from pathogenic *E. coli* and thus naïve to the strain used in this study. Further research is clearly needed to investigate to which extent the lack of prior exposure affects host responses of mice in infection models.

In summary, we present here a thorough analysis of host-pathogen interaction in a murine whole-blood model, the first point of fungal interaction with the host immune system in experimental candidiasis, and provide time-resolved data on fungal survival and association to immune cells. Our results indicate substantial differences to the host-fungal interactions shown in a human whole-blood model (summarized in **Table 1**), thereby limiting the translatability of data obtained in a murine whole-blood model to human infections—even though the murine systemic infection model recapitulates several important aspects of systemic infection in humans. This could be at least in part due to the lack of *C. albicans* colonization of SPF mice.

**Table 1:** Summary of the key differences between the murine and human whole-blood model for *C. albicans*.

	Murine whole blood	Human whole blood
***C. albicans* killing**	Not detectable	≥50% within 60 min, ≥80% within 180 min (4)
**Association *C. albicans* with leukocytes**	Approx. 40–60% max. (mouse strain-dependent)	Approx. 80% max (4).
**Association with neutrophils relative to all leukocytes**	≥50% (time point-dependent)	≥80% (4)
** Association with monocytes relative to all leukocytes**	≥25% (time point-dependent)	≤15% (4)
** Association with lymphocytes relative to all leukocytes**	≥50% (time point-dependent)	Not detectable (4)
**% association neutrophils with *C. albicans***	40–50% (mouse strain-dependent)	Not determined
**Filamentation (% hyphae)**	≥60% after 60 min; ≥ 90% after 180 min	≤10% after 60 min; ≤20% after 180 min
**Interaction with platelets**		
***C. albicans* association with platelets in whole blood**	≤60% after 10 min; 40% after 180 min	≤10%

## Data Availability Statement

The original contributions presented in the study are included in the article/**Supplementary Material**. Further inquiries can be directed to the corresponding author.

## Ethics Statement

The studies involving human participants were reviewed and approved by Ethics Committee of the University Hospital Jena, permit number: 273-12/09. The patients/participants provided their written informed consent to participate in this study. The animal study was reviewed and approved by Thüringer Landesamt für Verbraucherschutz, permit number 03-007/13 and HKI-19-003.

## Author Contributions

SM, SS, IJ, BS-R, OK, KH, and CS conceived the study. SM, SS, CD, KS, WK, KH, CS, and GR performed the experiments. SM, KH, CS, and IJ analyzed the data. SM, CD, and IJ drafted the manuscript. SM, SS, KH, OK, CD, KS, WK, BS-R, CS, GR, and IJ revised and approved the manuscript. All authors contributed to the article and approved the submitted version.

## Funding

Part of this project was funded by the funding line Strategic Networking in the Leibniz Association within the framework of the Leibniz Science Campus “InfectoOptics” (Project BLOODi to IJ) in Jena. CS was supported by the FWF Austrian Science Fund (Project Nr. P26117-B20). Work in the lab of OK and IJ was supported by the German Research Foundation (DFG; TRR 124 FungiNet, “Pathogenic fungi and their human host: Networks of Interaction,” DFG project number 210879364, Project C3 to OK and C5 to IJ). Funders had no role in study design, analyses and interpretation of data, in the writing of the report, and in the decision to submit the article for publication.

## Conflict of Interest

The authors declare that the research was conducted in the absence of any commercial or financial relationships that could be construed as a potential conflict of interest.
