# Supplementary material for: Significant Differences in Host-Pathogen Interactions Between Murine and Human Whole Blood
Source: Front Immunol. 2021 Jan 15;11:565869. doi: 10.3389/fimmu.2020.565869 (PMC7843371; doi:10.3389/fimmu.2020.565869)
Supplement: Supplementary file 1 [file DataSheet_1.pdf]

Supplementary Material

1 Supplementary Figures

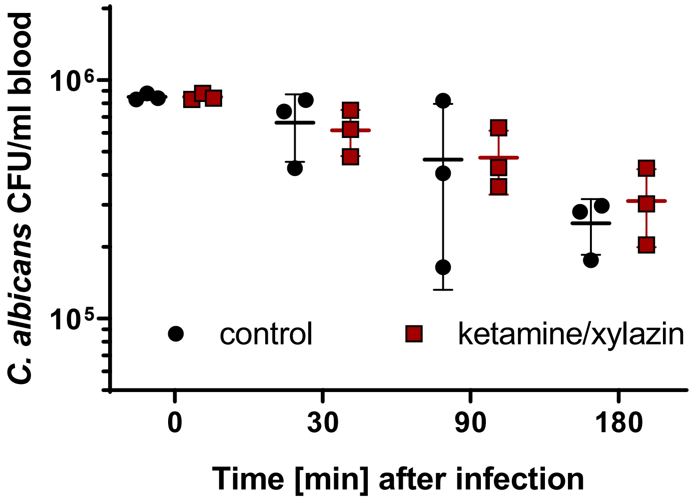

Supplementary Figure 1. Effect of ketamine and xylazin on *C. albicans* killing in the human whole-blood model. Blood from healthy human volunteers was left untreated (control, black circle) or 40 µg/ml ketamine and 2 µg/ml xylazin (ketamine/xylazin, red square) were added just before infection with *C. albicans*. Samples collected at the indicated time points were plated in serial dilution and CFU were quantified. Three experiments using different donors were performed with the control and ketamine/xylazin treatment performed in parallel. Mean ± standard deviation are indicated by the lines. Statistical analysis was performed by paired, two-tailed t test comparing control and ketamine/xylazin for each time point and showed no significant difference between the groups.

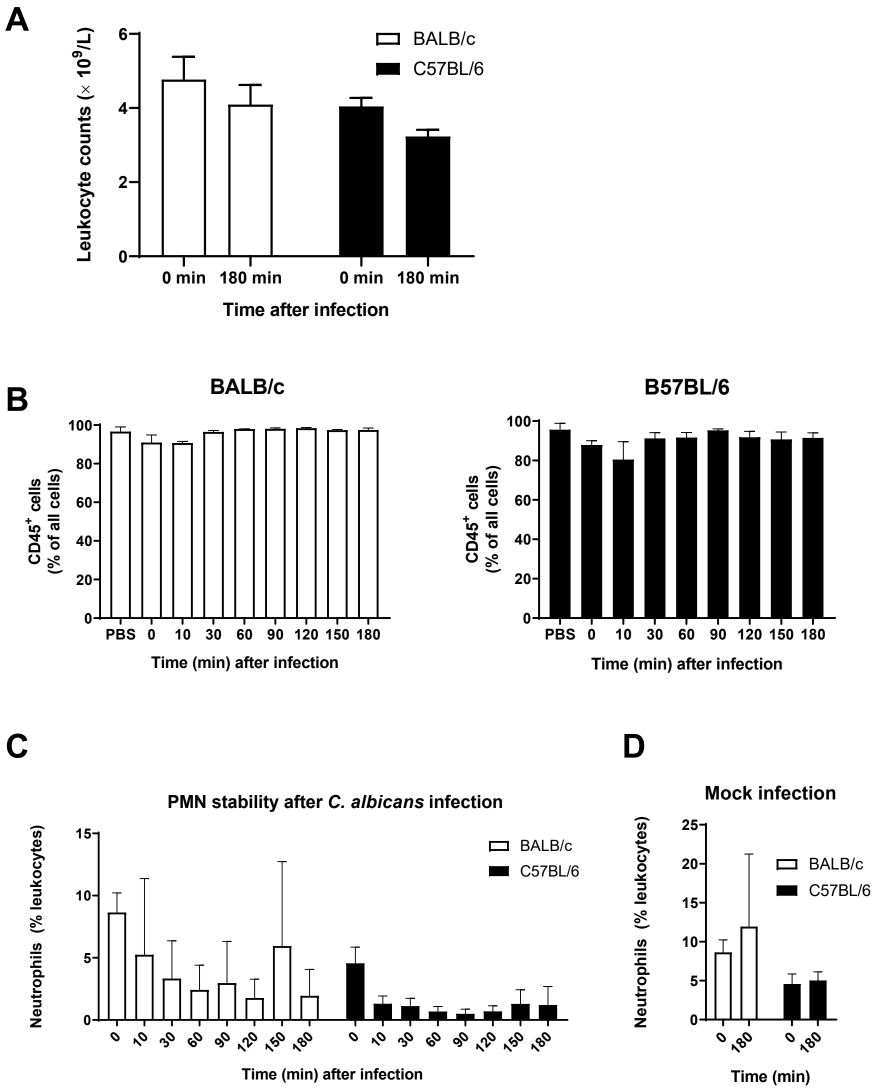

Supplementary Figure 2. Immune cell numbers in infected murine whole blood. (A) Total leukocyte counts were determined with an automated hematology analyzer. Changes over time were not statistically significant (unpaired t test corrected for multiple comparisons using the Holm-Sidak method). (B) The relative numbers of $CD45^+$ cells in blood were measured by flow cytometry. (C, D) Relative numbers of neutrophils ($Ly6G^+/CD11b^+$) in infected blood (C) and blood mock-infected with PBS (D) measured by flow cytometry. Data from three independent experiments is presented as mean ± standard deviation in each graph.

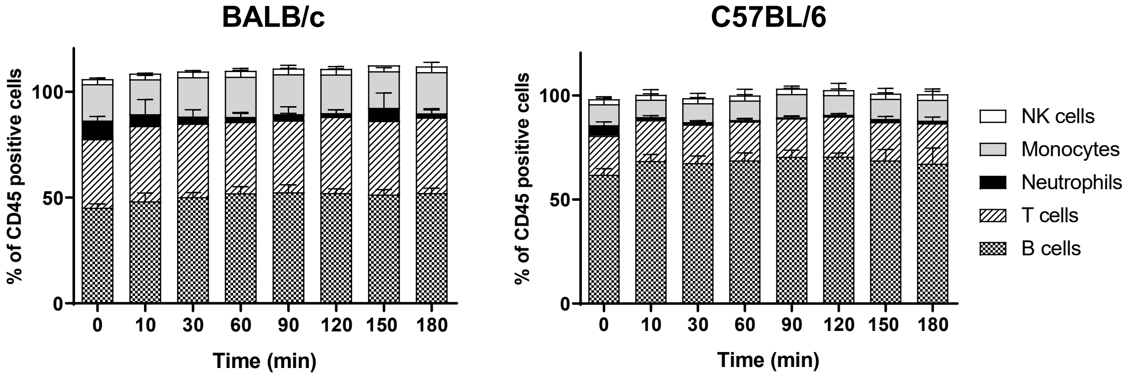

Supplementary Figure 3. Immune cell stability in murine whole blood upon exposure to *Candida*. Cell populations were measured by flow cytometry following *ex vivo C. albicans* infection of blood from BALB/c (A) and C57BL/6 mice (B). Statistical analysis was performed by unpaired, two-tailed t test.

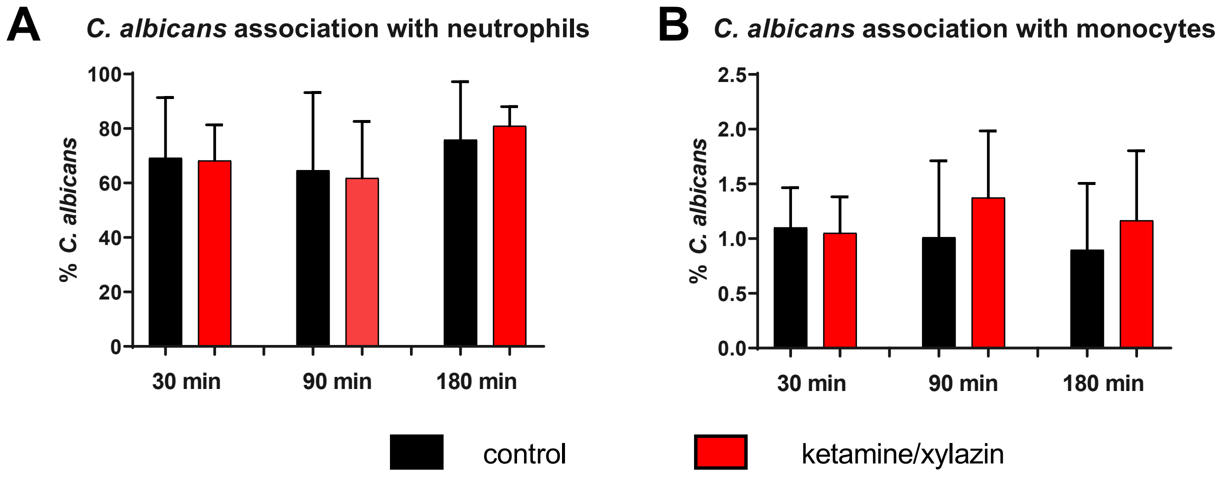

Supplementary Figure 4. Effect of ketamine and xylazin on association of *C. albicans* with neutrophils and monocytes in the human whole-blood model. Blood from healthy human volunteers was left untreated (control, black) or 40 µg/ml ketamine and 2 µg/ml xylazin (ketamine/xylazin, red) were added just before infection with *C. albicans*. Samples collected at the indicated time points were analyzed by flow cytometry. Three experiments using different donors were performed with the control and ketamine/xylazin treatment performed in parallel. The percentage of GFP-expressing *Candida* binding to immune cells of blood was calculated relative to total *C. albicans* cells in infected blood (set to 100 %) during a time course of three hours and is depicted as mean ± standard deviation. Statistical analysis was performed by paired, two-tailed t test comparing control and ketamine/xylazin for each time point and showed no significant difference between the groups.

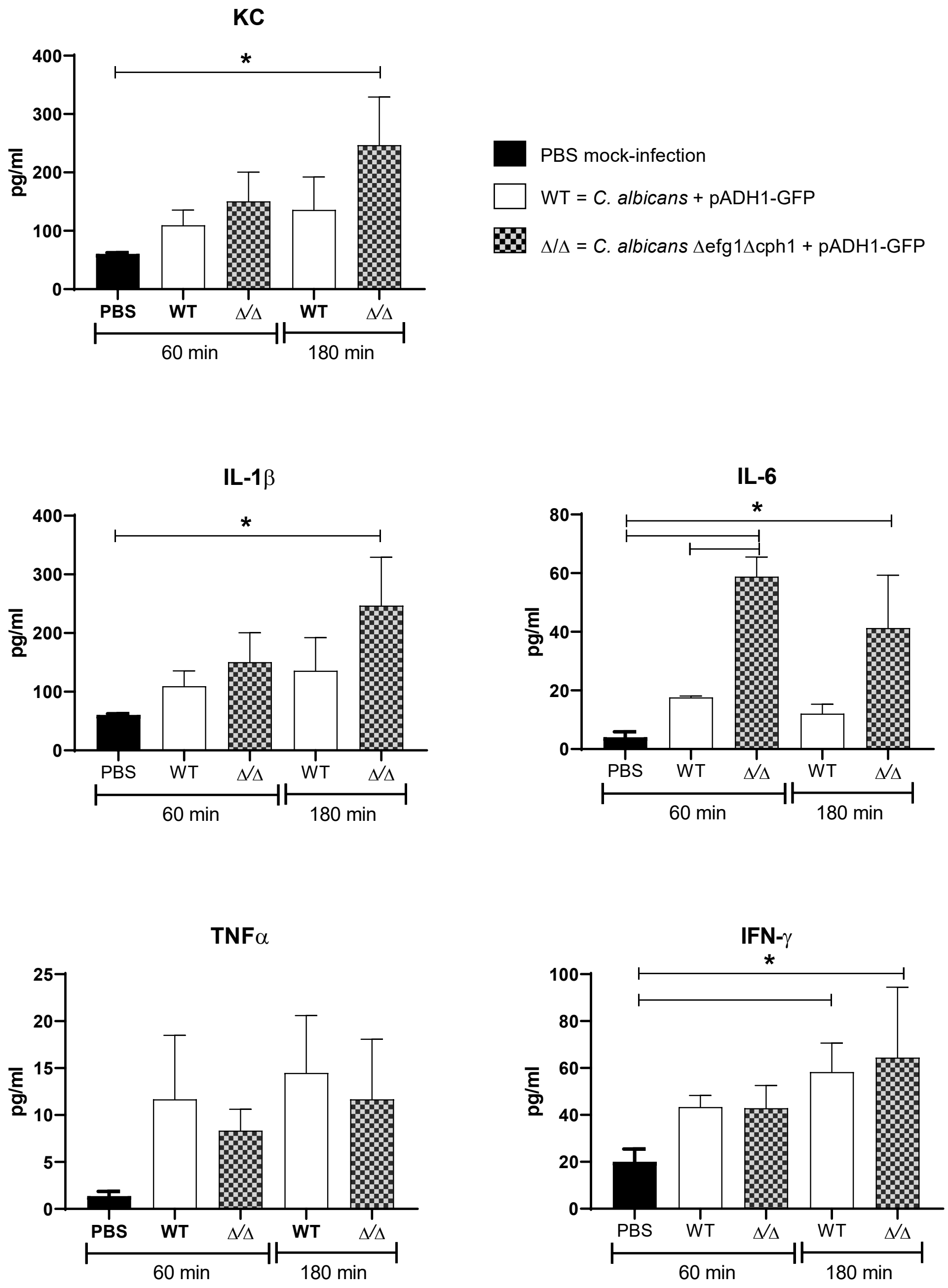

Supplementary Figure 5. Cytokine response to *C. albicans* infection in murine blood. Blood from BALB/c mice was infected with GFP-expressing *C. albicans* wild type (white) or *efg1*Δ*cph1*Δ double mutant (grey, square pattern). PBS-mock infection served as control. Cytokines were quantified in plasma at the indicated time points. Asterisks indicate significant differences ($p<0.05$; 1-Way ANOVA and Holm-Sidak's multiple comparison test for comparison of infected to control blood; unpaired, two-tailed t test for comparison of the two strains at one time point). Mean ± standard deviation of two to three independent experiments are shown.

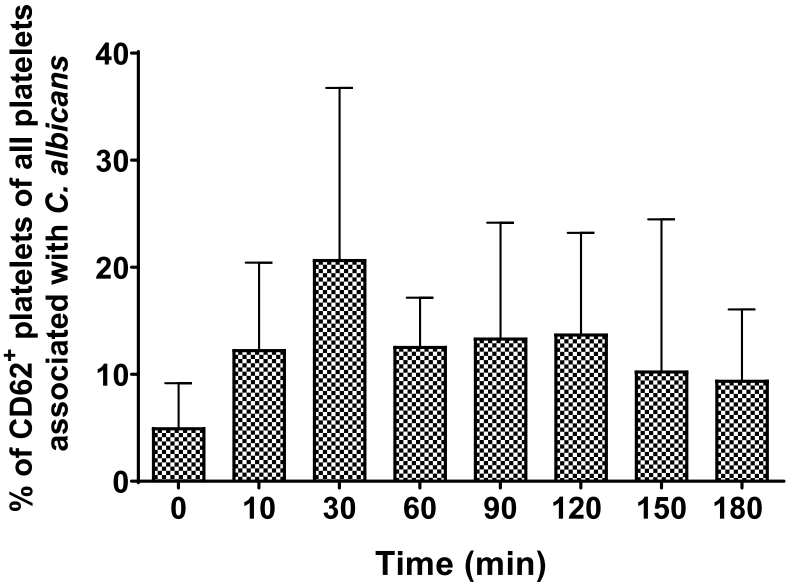

Supplementary Figure 6. Numbers of CD62-activated platelets associated with *C. albicans* during the time of infection. Cells were differentially stained in whole blood and activated platelets ($CD41^+$ $CD62^+$) that were also positive for GFP were included in the analysis. Activated platelets are shown in relative amounts to all positive platelets ($CD41^+$).

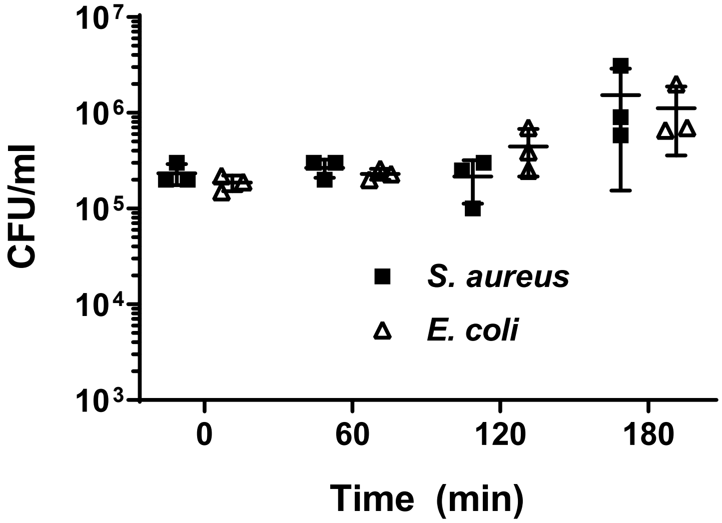

Supplementary Figure 7. Bacterial survival in murine whole blood at lower infection dose. Colony forming units of *S. aureus* and *E. coli* were determined from infected murine blood. All values correspond to the means of three independent experiments with pooled whole-blood from 10 BALB/c mice infected *ex vivo* with 1 x 10^5/ml of GFP-expressing bacteria. Each dot represents the mean of an independent experiment +/- standard deviation (n=3).
